# Lesion human leukocyte antigen-F expression is associated with a poor prognosis in patients with hepatocellular carcinoma

**DOI:** 10.3892/ol.2014.2686

**Published:** 2014-11-07

**Authors:** YONGFU XU, HAIXIONG HAN, FABIAO ZHANG, SHANGDONG LV, ZHENGYU LI, ZHEPING FANG

**Affiliations:** Department of Hepatobiliary Surgery, Taizhou Hospital of Zhejiang Province, Wenzhou Medical University, Linhai, Zhejiang 317000, P.R. China

**Keywords:** human leukocyte antigen-F, hepatocellular carcinoma, prognosis

## Abstract

Human leukocyte antigen (HLA)-F, a non-classical HLA-class I molecule, has attracted attention as an important immunosuppressive molecule in recent years, although the clinical relevance of HLA-F expression in cancer patients remains unclear. In the present study, HLA-F expression in 90 primary hepatocellular carcinoma (HCC) lesions and 55 corresponding adjacent normal liver tissues was analyzed by immunohistochemistry, and the associations between HLA-F expression and clinicopathological parameters and patient survival times were analyzed. Positive HLA-F expression was observed in 47.8% (43/90) of the HCC lesions and in 10.9% (6/55) of the normal liver tissues. HLA-F expression in HCC lesions was significantly correlated with patient gender (P=0.02), and venous or lymphatic invasion (P=0.02). Patients who were HLA-F-positive had worse survival times than those who were HLA-F-negative (P=0.04). The mean overall survival times for HLA-F-negative and -positive patients were 44.2 months [95% confidence interval (CI), 37.7–50.7] and 33.0 months (95% CI, 25.1–40.8), respectively. Multivariate analysis revealed that HLA-F was an independent prognostic factor for HCC patients with a hazard ratio of 2.1 (95% CI, 1.0–4.4). In conclusion, the present study demonstrated that HLA-F expression was associated with poor survival in HCC patients, and is correlated with tumor cell invasion and metastasis.

## Introduction

Hepatocellular carcinoma (HCC) is the fifth most prevalent form of cancer and the third leading cause of cancer-associated mortality worldwide (1). Tumor cells escape from immune-cell recognition and antitumor immune responses using numerous strategies (2). Alterations in human leukocyte antigen (HLA) expression, including HLA total loss, HLA haplotype loss, HLA-specific locus downregulation, HLA allelic losses and a combination of these phenotypes, are mechanisms widely used by tumor cells, and are critical in the development and progression of various types of malignancy (3). The non-classical HLA-class I molecules, including HLA-E, HLA-G and HLA-F, function as potential immunosuppressive molecules, directly or indirectly interacting with various types of immune cell, including natural killer (NK) cells, T cells, monocytes, macrophages and dendritic cells, to achieve immunosuppression (4,5). A number of studies have revealed alterations in HLA-G and HLA-E expression in >30 types of malignant tumor, including ovarian cancer, breast cancer, colon cancer, pituitary tumors and leukemia, and these changes were associated with poor patient survival (6). Recently, HLA-F has been widely investigated. Lepin *et al* (7) demonstrated that HLA-F/β2-tetramers bind to the immune inhibitory receptor immunoglobulin-like transcripts (ILT)-2 and ILT-4, indicating a potential role for HLA-F in the regulation of immune cell functions. A study by Zhang *et al* (8) indicated that the HLA-F^*^01:04 allele is associated with the risk of HCC pathogenesis. Furthermore, Noguchi *et al* (9) reported that anti-HLA-F IgG antibodies were present in sera derived from HCC patients.

However, thus far, no studies have been conducted with regard to the clinical relevance of HLA-F expression in HCC. In the present study, HLA-F expression in HCC was analyzed by immunohistochemistry, and its correlation with clinicopathological parameters and patient outcome were evaluated.

## Patients and methods

### Patients and specimens

A total of 90 primary tumor lesions and 55 case-matched adjacent normal liver tissue samples were consecutively collected from HCC patients undergoing curative resection at Taizhou Hospital of Zhejiang Province, Wenzhou Medical University (Linhai, China) between September 12, 2005 and October 12, 2011. A total of 78 male and 12 female patients with a median age of 53 years (range: 12 years to 74 years) were enrolled on this study. None of the patients had received preoperative radiotherapy, chemotherapy or any other medical intervention. The clinicopathological findings were determined according to the World Health Organization criteria (10) and the seventh edition of the tumor-node metastasis (TNM) classification of the American joint committee on cancer (11). The patient data collected included information regarding age, gender, tumor diameter, lymphatic or venous invasion, clinical tumor stage, date of initial diagnosis, and the date of fatality from HCC or the date of the last follow-up. Among the patients, 62.2% (56/90) were diagnosed with TNM stage I, 7.8% (7/90) were TNM stage II, 30.0% (27/90) were TNM stage III and no case was stage IV. Of the 90 cases, 56 were suitable for follow-up. The follow-up period was 60 months or until the patient succumbed to the disease. The average follow-up for all patients was 33.6 months (range, 8–60 months) and during the entire period, 30 cancer-associated fatalities (53.6%) were recorded. The study was performed after the Ethics Review Board of Taizhou Hopsital of Zheijiang Province approved the study procedure to investigate the molecular markers associated with HCC pathogenesis and informed consent was obtained from all patients.

### Immunohistochemistry and staining evaluation

Immunohistochemistry was performed according to standard methods as previously described (12). The 14670-1-AP rabbit polyclonal anti-human HLA-F antibody (1:300; Proteintec Group, Chicago, IL, USA) was used to probe for the expression of HLA-F overnight at 4°C. Goat polyclonal polyperoxidase anti-mouse and anti-rabbit IgG (Dako, Glostrup, Denmark) secondary antibody was then applied for 30 min at 37°C. Diaminobenzidine solution was used as a chromogen. Finally, sections were counterstained with hematoxylin and mounted with glycerol gelatin (Zhongshan Biological Technology Co., Ltd., Beijing, China). The extent of HLA-F staining in the HCC tissues was determined by three independent pathologists, who were blinded to the clinical data and the disease outcome. The percentage of HLA-F-positive tumor cells was assessed by each observer and the average of the scores was recorded as the final result. A lesion was scored as positive when the percentage of HLA-F-positive tumor cells in the entire lesion was >5% and negative when the percentage was ≤5%. Both membrane and cytoplasmic expression of HLA-F were interpreted as positive. The percentage of positive cells was assigned a value as determined by the presence or absence of HLA-F staining, regardless of the staining intensity.

### Statistical analysis

Statistical analysis was performed using SPSS 17.0 software (SPSS, Chicago, IL, USA). Correlations between HLA-F expression and clinical parameters were calculated using the Pearson χ^2^ test. The overall patient survival time was calculated as the time period between the date of diagnosis and the date of last follow up (censored) or date of patient mortality (event). The survival probabilities were determined using the Kaplan-Meier method and statistical significance was calculated using the log-rank test. The correlations between survival time and multiple clinicopathological variables in univariate and multivariate analysis were calculated using Cox regression analysis. P<0.05 was considered to indicate a statistically significant difference.

## Results

### HLA-F expression in primary HCC lesions and normal liver tissues

**Figure 1:**
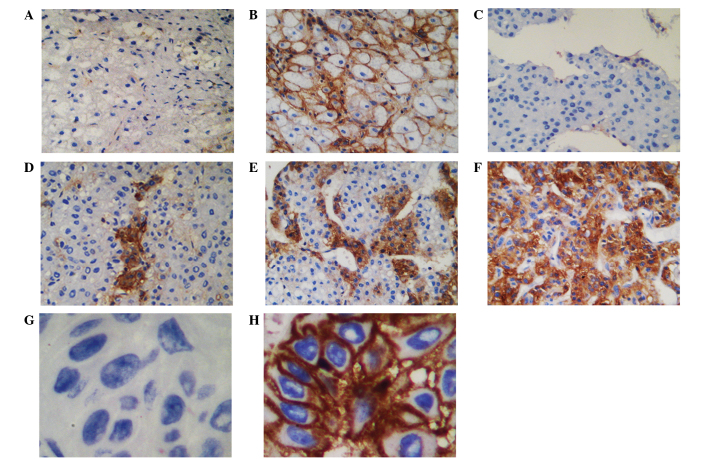
Immunohistochemical staining of human leukocyte antigen-F (HLA-F) expression in hepatocellular carcinoma (HCC) lesions and normal liver tissues. Immunohistochemically (A) negative and (B) positive expression of HLA-F in normal liver tissues. (C) Negative, (D and E) ≤50% positive and (F) >50% positive HLA-F expression in HCC lesions. HLA-F expression was considered as negative when the percentage of stained cells was ≤5%. Original magnification, ×100. (G) Negative and (H) positive skin cancer lesion controls. Original magnification, ×400.

The membrane and cytoplasmic expression of HLA-F in the specimens was determined through immunohistochemical staining. The intensity of staining varied among tumors and among tumor areas within the same specimen. Heterogeneous staining was observed in all HLA-F-positive HCC lesions. Lesions from skin cancer patients treated at Taizhou Hopsital of Zhejiang Province served as internal positive and negative (with isotype IgG1) controls for HLA-F expression. HLA-F expression was observed in 47.8% (43/90) of the HCC lesions and in 10.9% (6/55) of the normal liver tissues (Fig. 1; χ^2^=20.741, P<0.05).

### HLA-F expression in HCC lesions relative to clinicopathological parameters

The data revealed that venous or lymphatic invasion (χ^2^=5.388, P=0.020), and patient gender (χ^2^=5.371, P=0.020) were significantly associated with positive HLA-F expression. However, no significant differences in HLA-F expression were observed between other clinical parameters, such as patient age (χ^2^=0.156, P=0.693), tumor diameter (χ^2^=0.002, P=0.962) and TNM stage (χ^2^=0.584, P=0.445) (Table I).

### HLA-F expression is associated with survival times in HCC patients

**Figure 2:**
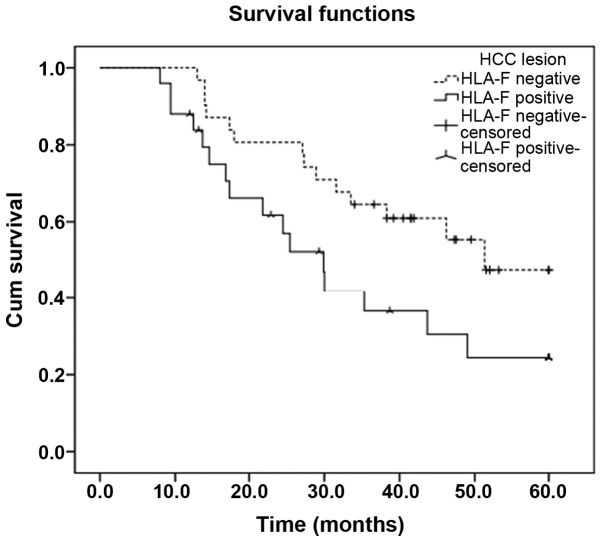
Kaplan-Meier survival analysis of human leukocyte antigen-F (HLA-F) expression in hepatocellular carcinoma (HCC) patients. Comparison of patient overall survival times of HLA-F-negative (n=31) and -positive patients (n=25, P=0.040).

Patient survival times were defined as the duration from the date of diagnosis to the date of death. Patients with HLA-F-positive primary tumors exhibited significantly shorter survival times than patients with HLA-F-negative tumors (χ^2^=4.210, P=0.04; Fig. 2). The mean survival time of the HLA-F-positive HCC patients was 33.0 months [95% confidence interval (CI), 25.1–40.8 months], which was significantly shorter than that of the HLA-F-negative HCC patients [44.2 months (95% CI, 37.7–50.7 months)] (P=0.040; Fig. 2). In addition, Cox proportional-hazards model analysis was performed to assess the prognostic parameters in patients with HCC. In the univariate analysis, HLA-F-positive expression (HR, 3.061; P=0.012) and TNM stages II/III (HR, 1.632; P=0.033) had significantly higher hazard ratios than HLF-A-negative expression and TNM stage I, respectively, indicating a poor prognosis. Furthermore, multivariate analysis revealed that positive HLA-F expression was an independent prognostic factor (HR, 2.149; P=0.039; Table II).

## Discussion

In the present study, HLA-F was observed to be more frequently expressed in HCC lesions than in normal adjacent tissue sections. Notably, HLA-F expression was significantly correlated with the degree of lymphatic or venous invasion. In addition, HLA-F expression was an independent prognostic factor for HCC patients.

HLA-F expression is exhibited in different manners in various types of tumor. In non-small-cell lung cancer (NSCLC) patients (12), HLA-F expression was detected in 24.1% (20/83) of NSCLC primary lesions but not in any of the adjacent normal lung tissues. Furthermore, HLA-F expression was not significantly associated with certain clinical parameters, such as patient age, gender, tumor histological type, tumor diameter, grade of tumor differentiation or TNM stage. However, patients with HLA-F-positive tumors had a significantly poorer prognosis than those who were HLA-F-negative; thus, HLA-F expression status was an independent prognostic factor for NSCLC patients. In esophageal squamous cell carcinoma (ESCC) patients (13), positive HLA-F expression was observed not only in tumor lesions (58.1%) but also in the corresponding adjacent normal esophageal tissues (54.8%). ESCC patients with HLA-F-positive tumors also had worse survival times than patients with HLA-F-negative tumors. Zhang *et al* (14) analyzed HLA-F expression in 277 primary gastric cancer (GC) lesions and HLA-F expression was observed in 71.1% (197/277) of the patients. The authors found that lesion HLA-F expression was not associated with clinical parameters, such as gender, age or disease TNM stage and, unlike in NSCLC and ESCC patients, HLA-F expression was not associated with GC patient prognosis, and therefore may exert a cancer-type dependent effects.

In the present study, the HLA-F expression in the HCC lesions was significantly correlated with the lymphatic or venous invasion. Ishigami *et al* (15) also demonstrated that HLA-F expression in GC lesions was significantly associated with lymphatic and venous invasion, as well as depth of invasion and nodal involvement. Thus, HLA-F expression may be associated with aggressive tumor behavior, and the promotion of tumor cell invasion and metastasis. However, further studies are required to investigate this hypothesis.

HLA-F, which is encoded by a gene located on the short arm of human chromosome 6, was first identified by Geraghty in 1990. The HLA-F protein is 5.4 kb in length and is highly homologous with other types of HLA-I molecule (16,17). HLA-F is shorter than a typical HLA class I protein due to the exclusion of exon 7 from the mature HLA-F mRNA, resulting in a protein with a shortened cytoplasmic domain (16). HLA-F was found to be entirely dependent on its cytoplasmic tail for export from the endoplasmic reticulum, with the assistance of the C-terminal valine residue and the RxR motif (18). However, whether HLA-F is expressed on the cell surface remains controversial. Early studies indicated that HLA-F exhibits predominantly intracellular expression, although this was only observed in the cytoplasm of peripheral blood B cells, B cell lines, tissues containing B cells, and various other types of tissues and cell lines, such as bladder and skin cell lines (7,19). Later studies observed that HLA-F protein may be expressed at the surface of certain cell types; for example, EBV-transformed lymphoblastoid cell lines or particular monocyte cell lines, extravillous trophoblast cells invading the decidua in term placental tissues, and activated B cells, T cells, NK cells and monocytes (20–22). The present study revealed both membrane and cytoplasmic expression of HLA-F.

Studies concerning the function of HLA-F have rarely been reported. A previous study (7) demonstrated that HLA-F tetramers were capable of interacting with the inhibitory receptors ILT-2 and ILT-4, indicating a mechanism by which HLA-F exerts an immune tolerance function. Goodridge *et al* (23) revealed that HLA-F and the open conformers of major histocompatibility complex I (MHC-I) expressed on activated cells cooperate in a MHC-I antigen cross-presentation pathway for the presentation of exogenous proteins by MHC-I, which may significantly contribute to the regulation of immune system functions and immune defense. HLA-F has also been shown to be involved in maternal-fetal tolerance. Shobu *et al* (24) indicated that HLA-F may function together with HLA-G/E to prepare an environment to support fetal growth. We hypothesize that HLA-F expression may also enable tumor cells to escape from recognition by the host immune system.

In conclusion, in the present study, positive HLA-F expression was associated with poor survival in HCC patients, and may be correlated with invasion and metastasis of tumor cells. This finding provides a novel area for the analysis of HCC. However, the biological functions and clinical significance of HLA-F are far from established, and further investigation into HLA-F expression is urgently required.

## Figures and Tables

**Table I:** Association of HCC lesion HLA-F expression with patient clinicopathological parameters^a^.

		HLA-F expression			
		
Variable	No. of cases	Negative (%)	Positive (%)	χ^2^	P-value
Total	90	47 (52.2)	43 (47.8)		
Gender				5.371	0.020
Male	78	37 (47.4)	41 (52.6)		
Female	12	10 (83.3)	2 (16.7)		
Age (years)				0.156	0.693
≤53	48	26 (54.2)	22 (45.8)		
>53	42	21	21		
T factor (cm)				0.002	0.962
≤5	50	26 (52.0)	24 (48.0)		
>5	40	21 (52.5)	19 (47.5)		
V/Ly factor				5.388	0.020
Yes	72	42 (58.3)	30 (41.7)		
No	18	5 (27.7)	13 (72.3)		
TNM stage				0.584	0.445
I	56	31 (55.4)	25 (44.6)		
II/III	34	16 (47.1)	8 (52.9)		

a Comparison of HLA-F expression status between or among variables was performed using the Pearson χ^2^ test.

HCC, hepatocellular carcinoma; HLA-F, human leukocyte antigen-F; T factor, tumor diameter; V/Ly factor, venous or lymphatic invasion; TNM, tumor, node, metastasis.

**Table II:** Cox proportional-hazards model analysis of variables affecting survival rates in HCC patients^a^.

		Overall survival rate			
		
		Univariate analysis		Multivariate analysis	
			
Variable	Category	HR (95% CI)	P-value	HR (95% CI)	P-value
Gender	Male (vs. female)	2.024 (0.576–7.108)	0.271		
Age (years)	>53 (vs. ≤53)	0.504 (0.207–1.225)	0.130		
T factor (cm)	>5 (vs. ≤5)	1.871 (0.893–3.919)	0.097		
V/Ly factor	Yes (vs. no)	0.744 (0.247–2.246)	0.600		
TNM stage	Stage II/III (vs. I)	1.632 (1.041–2.560)	0.033	1.558 (1.038–2.338)	0.032
Lesion HLA-F	Positive (vs. negative)	3.061 (1.285–7.295)	0.012	2.149 (1.040–4.441)	0.039

a Using Cox proportional-hazard analysis, multivariate models were covariate adjusted for TNM stage and lesion HLA-F expression.

HCC, hepatocellular carcinoma; HR, hazard ratio; 95% CI, 95% confidence interval; T factor, tumor diameter; V/Ly factor, venous or lymphatic invasion; TNM, tumor, node, metastasis; HLA-F, human leukocyte antigen.
